# Unbiased assessment of disease surveillance utilities: A prospect theory application

**DOI:** 10.1371/journal.pntd.0007364

**Published:** 2019-05-01

**Authors:** Arthur E. Attema, Lisheng He, Alasdair J. C. Cook, Victor J. Del Rio Vilas

**Affiliations:** 1 Erasmus School of Health Policy & Management, Erasmus University, Rotterdam, the Netherlands; 2 Department of Psychology, University of Pennsylvania, Philadelphia, PA, United States of America; 3 School of Veterinary Medicine, University of Surrey, Guildford, United Kingdom; 4 Centre on Global Health Security, Chatham House, London, United Kingdom; Oregon Health and Science University, UNITED STATES

## Abstract

**Objectives:**

We contribute a new methodological approach to the ongoing efforts towards evaluating public health surveillance. Specifically, we apply a descriptive framework, grounded in prospect theory (PT), for the evaluation of decisions on disease surveillance deployment. We focus on two attributes of any surveillance system: timeliness, and false positive rate (FPR).

**Methods:**

In a sample of 69 health professionals from a number of health related networks polled online, we elicited PT preferences, specifically respondents’ attitudes towards gains, losses and probabilities (i.e., if they overweight or underweight extreme probabilities) by means of a series of lotteries for either timeliness or FPR. Moreover, we estimated willingness to pay (WTP) for improvements in the two surveillance attributes. For contextualization, we apply our framework to rabies surveillance.

**Results:**

Our data reveal considerable probability weighting, both for gains and losses. In other words, respondents underestimate their chances of getting a good outcome in uncertain situations, and they overestimate their chances of bad outcomes. Moreover, there is convex utility for losses and loss aversion, that is, losses loom larger than gains of the same absolute magnitude to the respondents. We find no differences between the estimated parameters for timeliness and FPR. The median WTP is $7,250 per day gained in detection time and $30 per 1/10,000 reduction in FPR.

**Conclusion:**

Our results indicate that the biases described by PT are present among public health professionals, which highlights the need to incorporate a PT framework when eliciting their preferences for surveillance systems.

## Author summary

In this paper we contribute a new methodological approach to the ongoing efforts towards evaluating public health surveillance. Specifically, we apply a descriptive framework for the evaluation of decisions on disease surveillance deployment. We focus on two attributes of any surveillance system: timeliness and false positive rates. In a sample of 69 health professionals from a number of health related networks polled online, we elicited preferences, specifically respondents’ attitudes towards gains, losses and probabilities (i.e., if they overweight or underweight extreme probabilities) by means of a series of lotteries. For contextualization, we apply our framework to rabies surveillance. Our data reveal that respondents underestimate their chances of getting a good outcome in uncertain situations, and they overestimate their chances of bad outcomes. Moreover, losses loom larger than gains of the same absolute magnitude to the respondents. We find no differences between the estimated parameters for timeliness and false positive rates. Our results indicate that the biases described are present among public health professionals, and highlight the need to adjust for them when eliciting their preferences for surveillance systems.

## Introduction

Disease surveillance system (DSS) evaluation is a topical issue that has led to the development of multiple frameworks and methods. While differences remain among these methods, most contain a number of criteria or attributes to capture the multi-dimensional nature of the challenge and to qualify the performance of the DSS [1]. Sensitivity (the capacity of the DSS to detect and quantify the event of interest), timeliness (the capacity to detect the event within pre-defined times), and false positive rate (FPR) (related to the capacity of the DSS to truly detect the events of interest; in other words informing the efficiency of the DSS) are common criteria across many frameworks [2]. The last two are the target of this work.

Improvement (deterioration) in timeliness and FPR deliver some level of utility (disutility), understood as the satisfaction or usefulness derived from the materialization of those attributes, to the relevant surveillance stakeholders. The level of utility (disutility) delivered may vary between stakeholders, and even for the same stakeholders facing different contexts. For example, in a gains setting context, where increased surveillance investments may lead to improved timeliness and FPR vs. a loss setting context with surveillance de-investments possibly leading to inferior timeliness and FPR. Assessment of this utility (disutility), adjusted for known biases (see below), should be an integral part of any surveillance evaluation.

Rabies is a neglected disease, fatal in most cases once clinical signs appear, and the alleged cause of more than 50,000 deaths/year [3]. Rabies is also entirely preventable with the administration of timely and adequate post-exposure-prophylaxis (PEP). Despite the significant death toll, rabies surveillance remains under-developed and lacks systematic approaches to its assessment [4]. The need to address human rabies surveillance limitations becomes urgent given the 2030 global elimination goal, and the specific requirement of an “effective surveillance” for validation and certification of disease freedom [5]. Of critical relevance for an effective rabies surveillance is the timely detection of exposed cases to allow immediate implementation of PEP, in an efficient manner, i.e. with as few false positive exposures as possible in order to limit PEP wastage (that is, a low FPR). PEP is an expensive resource and its availability limited, most so in developing settings where rabies remains endemic [6,7]. A number of works have reported the excessive prescription of PEP in different settings [8] and the need to optimize its administration to facilitate the sustainability of rabies programs.

This work aims to improve the understanding of the underlying attitudes and motivations for/against the investment of resources in enhanced surveillance approaches. We focus on enhancements leading to improvements in rabies surveillance timeliness and false positive rate. Our work applies a valid descriptive framework, grounded in prospect theory (PT) [9], to quantify the effects of several cognitive biases, namely, loss aversion and probability weighting, and the relevance of the reference point (a proxy for stakeholders’ context) for the utility of surveillance timeliness and false positive rate. Briefly, PT is a descriptive theory that explains decisions under risk and describes several cognitive biases. First, people tend to form reference points (RP’s) and regard outcomes as deviations from this RP. Hence, people are sensitive to *changes* in outcomes rather than to *final* outcomes. The relevance of RP to properly frame interventions to increase their acceptability and uptake is well established in healthcare settings [10]. Second, people make a distinction between outcomes above the RP (gains) and outcomes below it (losses). They perceive losses to loom much larger than gains of the same absolute magnitude, which results in a higher weight being attached to losses than to gains. This phenomenon is known as *loss aversion* and can influence the uptake of interventions. Third, people have difficulties to process probabilities, which they transform nonlinearly into decision weights. This behavior is called *probability weighting* and often causes small probabilities to be overweighted, and large probabilities to be underweighted. The purpose of the study is to evaluate the presence of these cognitive biases in surveillance investment decisions and to quantify their magnitude, in order to disentangle the different components of risk aversion. This is critical to compute unbiased utilities associated with surveillance or disease control interventions, which inform strategic settings. To the best of our knowledge, the application of PT in a surveillance setting is novel.

## Methods

### Ethical statement

No ethical approval was required for our study. All study participants gave informed consent.

### General approach

We polled members of different public health and animal health networks/communities to inform the aforementioned PT parameters. S1 Raw data were collected by means of an online questionnaire, which was programmed in Qualtrics. The full questionnaire can be found in the supplementary materials. For both the timeliness and FPR attributes, the survey consisted of four different parts. The first three parts were used to elicit the utility of gains, the utility of losses, and loss aversion (detailed below), respectively. The last part of the questionnaire aimed to elicit respondents’ willingness to pay (WTP) for an improvement in timeliness and FPR, respectively. In addition, we elicited the respondents’ RPs. Each part was introduced with a brief description of the choice situation, followed by a practice question. The different parts were presented in randomized order.

We apply (cumulative) prospect theory with specific functional forms for utility and probability weighting [11–13]. For the utility functions for gains and losses, we assume the commonly used power function, and for probability weighting we estimate Prelec’s [14] one-parameter model. Loss aversion is defined as the ratio of the slopes of the loss and gain utility functions [15], and preferences are elicited by means of comparing risky options to certain options. Indifferences are obtained by the use of choice lists, which is the most common technique to elicit risk preferences in economics [16,17].

### Terminology and model

We consider preferences over two-outcome lotteries (x,p;y,1-p), which give outcome x with probability p (0<p<1) and outcome y with probability 1-p. Furthermore, we assume preferences are reference-dependent with respect to a reference point r. Gains are outcomes that are strictly preferred to r and losses are outcomes strictly less preferred to r. A gain prospect involves no losses, a loss prospect involves no gains, and a mixed prospect involves both a gain and a loss.

In this study, we assume subjects behave according to prospect theory. In particular, we assume two common parametric shapes of the utility function and the probability weighting function. For utility, we assume the usual power function:

                                                      *U*(*x*) = *x*^*α*^ for gains and:

$$U(x)=-{\left(-x\right)}^{\beta }\;\mathrm{for}\;\mathrm{losses}.$$ (1)

With U(x) the utility of outcome x, α,β>0, and α,β<1 implying a concave [convex] utility for gains [losses]. Similarly, α,β>1 implies a convex [concave] utility for gains [losses]. α,β = 1 implies linear utility.

Probability weighting is modeled according to Prelec’s [14] one-parameter function:

$$w^{i}(p)=\exp\left\{-{\left(-\ln(p)\right)}^{j}\right\},$$ (2)

with *w*^*i*^(*p*) representing the decision weight given to probability p, i = +,- (i.e. we have separate weighting functions for gains and losses), and j = γ for gains and j = δ for losses. Whenever 0<j<1, this function has the familiar inverse S-shape, with overweighting of small probabilities and underweighting of large probabilities. In the case of surveillance, it means that health professionals would give too much weight to a small probability of improved timeliness and FPR, too little weight to higher probabilities, and that they are not sensitive enough to changes in this probability in the middle of the probability spectrum. This function also causes insensitivity to probabilities in the middle, and extreme sensitivity to changes from impossible to possible (e.g. a slight change from p = 0 to p = 0.01) and from possible to certain (e.g. from p = 0.99 to p = 1). Expected utility theory (i.e. no probability weighting) is a special case of this function when j = 1. Finally, loss aversion is modeled by multiplying the utility of losses by a factor λ. A decision maker is said to be loss averse if λ>1, gain seeking if λ<1, and loss neutral if λ = 1. In our context, loss aversion implies that health professionals would give too much weight to deteriorations in timeliness and the FPR compared to improvements in them. Appendix A gives a derivation of the regression equations that result from the aforementioned assumptions.

### Experiment

#### Subjects

In order to estimate the required sample size, we performed a power analysis using estimates of the standard deviation of the relevant parameters obtained by related studies in the monetary and health domains [11,18–21]. These studies indicated that the standard deviation of the estimated PT parameters were mostly below 0.10. In our study, we are mainly interested in testing for deviations from linearity in the utility function (i.e. significant difference from α,β = 1), from linear probability weighting (i.e. γ,δ = 1), and from loss neutrality (i.e. λ = 1). Second, we want to investigate if there are within-respondent differences in utility curvature and probability weighting between gains and losses (i.e. a test of α = β and γ = δ). According to Cohen’s *d* [22], a standardized effect size (i.e., the ratio of the effect size–any deviation from either side of 1 for each parameter–to the standard deviation) between 0.2 and 0.5 can be considered small. If we take the midpoint of this (0.35), we require a sample size of n = 67 to find such a small effect of 0.035 (i.e. 0.35×0.1) with a power of 0.80 and Type I error of 0.05.

Our subject pool consisted of a voluntary sample of 69 health professionals (44 women, 23 men, 2 not specified), 46 (66%) of whom had experience working with rabies (median years of experience: 3.25). Subjects were recruited from several networks either targeting rabies control (the Global Alliance for Rabies Control (GARC), the Middle East and Eastern Europe Rabies Expert Bureau (MEEREB), and Rabies in the Americas (RITA)), DSS (the International Society for Disease Surveillance (ISDS)), or One Health issues (the Network for Evaluation of One Health (NEOH), and Med-Vet-Net). Participants were recruited by email and did not get any reward for their participation. Random allocation assigned 41 subjects to the timeliness attribute and 28 to the FPR attribute. Respondents were from 24 different countries and most (27) from the United States of America.

#### Procedure

We used a choice list to elicit preferences, which is common in economic experiments. In short, a choice list consists of a number of binary choices on two options. One of the options remains fixed throughout the entire list, while the other becomes more (in the gains domain) or less (in the loss domain) attractive going down the list. When assessing the choices between the two options, subjects will tend to find the fixed option to be more attractive at top of the list. When moving further down the list, subjects will switch to the variable option at some point. The switching row then gives the two options that subjects consider to be about equally attractive (which is known as the “indifference point”). This indifference point gives an indication of the subject’s risk attitude.

#### Timeliness attribute

In the gain part, respondents were asked to imagine an area with endemic dog-mediated rabies and a passive human surveillance system with an average time to detection of rabies suspect exposures (i.e. people bitten by suspect rabid dogs) of 20 days. There were two possible alternatives to this surveillance system that could improve the time to detection. One option was a lottery that could improve the average time to detection by *x* days (e.g. 10 days) with probability *p* (in case of a successful intervention, e.g. 25%) but could also improve it by *y* days (e.g. 2 days, in case the intervention turns out to be not so successful) with probability 1-*p* (e.g. 75%). The other option was a sure amount of improvement in the average time to detection (with 100% certainty).

We created eight choice questions for the gain part. This number was large enough to enable estimation of the parameters and small enough not to be too cognitively demanding. For each choice, respondents chose between the lottery and eight different sure gains. The sure gains lay between the worst and best possible outcomes in the lottery. The eight choice questions were organized in a list. An additional function was used to force participants to make consistent choices so that once a sure gain was selected, it would automatically select all sure gains larger than that particular sure gain. If the lottery was selected in a choice, the function would automatically choose the lottery in choices that had a smaller sure gain than the sure gain in that particular choice. Participants were allowed to change their choices by going backwards throughout the questionnaire.

We elicited certainty equivalents (CE’s) from these binary choices. CE’s describe the sure amount of outcome, timeliness or FPR in our case, that a subject would be willing to receive to be indifferent between that sure outcome and a given lottery. Particularly, for each question, a CE was estimated as the arithmetic mean of the largest sure gain that was turned down and the smallest sure gain that was preferred to the lottery. If all eight sure gains were chosen, the CE was estimated as the arithmetic mean of the smallest sure gain and the worst possible outcome of the lottery. If no sure gain was chosen, the CE was estimated as the arithmetic mean of largest sure gain and the best possible outcome of the lottery.

The loss part was the same as the gain part except that the two possible outcomes in the lotteries were worse than the status quo. Respondents were asked to imagine that due to budget cuts, the current system had to be replaced by some alternative that could lead to a deterioration in time to detection (a deterioration of timeliness). There were two replacement options. One option was a lottery that could lead to a deterioration of the average time to detection by *x* days (e.g. 10 days) with probability *p* (e.g. 25%) but could also lead to a deterioration of *y* days (e.g. 2 days) with probability 1-*p* (e.g. 75%). Eight lotteries were created and CE’s were estimated in the same way as for gains.

To elicit loss aversion, a mixed-prospect choice list was used. On the same scenario, with an existing DSS with an average time to detection of rabies suspect exposures (i.e. people bitten by suspect rabid dogs) of 20 days, the alternative surveillance was a lottery. The lottery could decrease the average time to detection by 10 days, or it could increase it by X, with even odds. The value of X varied across choices.

The fourth and last part of the questionnaire presented another choice list to elicit respondents’ WTP for a decrease in time to detection. The respondents were instructed that the current surveillance system had a timeliness of 20 days and cost $50,000. The alternative system had a timeliness of 10 days and cost $X. The value of $X that made the respondent indifferent between the two alternatives was the respondent’s WTP. Table 1 presents the lotteries offered to the respondents for the timeliness outcome. The combinations of probabilities and outcomes were chosen so as to cover the complete probability and outcomes spectrum as well as possible.

**Table 1:** Lotteries for the attribute/outcome “Timeliness”. Second and third column show the lotteries, probability of the outcome first and outcome (# of days to detection) second, presented to the respondents.

Number	Lotteries	
Losses		
1	0.25,-70	0.75,-7
2	0.4,-70	0.6,0
3	0.1,-70	0.9,-25
4	0.5,-70	0.5,-18
5	0.5,-35	0.5,0
6	0.75,-63	0.25,-14
7	0.5,-53	0.5,-11
8	0.25,-53	0.75,0
Gains		
9	0.25,20	0.75,2
10	0.4,20	0.6,0
11	0.1,20	0.9,7
12	0.5,20	0.5,5
13	0.5,10	0.5,0
14	0.75,18	0.25,4
15	0.5,15	0.5,3
16	0.25,15	0.75,0
Mixed		
	0.5,-X	0.5,10
WTP		
	20 days, $50,000	10 days, $X

Finally, we asked the respondents to report their own RPs. Respondents chose from options representing ranges of time to detection of suspect rabies exposures that would best match the time to detection of the rabies surveillance system of their own countries/areas. Alternatively, if they did not know the time to detection of their rabies surveillance system, they were instructed to choose the option for which they felt neutral, i.e. it was neither a gain nor a loss.

### FPR attribute

For the FPR attribute, the four parts were similar to those of the timeliness attribute. Respondents were asked to imagine a rabies surveillance system with an average annual FPR of 4,000 out of 10,000 suspect exposures. The FPR condition also consisted of eight choice lists in the gain part, eight choice lists in the loss part and one choice list in the mixed part. This prospect had to be traded off against the status quo, which was set to be 4,000/10,000.

The three parts were presented in a random order. Next we elicited the respondents’ WTP. The respondents were instructed that the current surveillance system had an FPR of 4,000/10,000 and costed $50,000. The alternative system had an FPR of 3,000/10,000 and costed $X.

Finally, we asked respondents for their own RPs. Respondents chose from options representing ranges of FPR that would best match the FPR of the rabies surveillance system of their own countries/areas. If they did not know the FPR of their rabies surveillance system, they were instructed to choose the option for which they felt neutral, i.e. it was neither a gain nor a loss. The stimuli of the FPR attribute are listed in Table 2.

**Table 2:** Lotteries for the attribute/outcome “False Positive Rate (FPR)”. Second and third column show the lotteries, probability of the outcome first and outcome (#/10,000) second, presented to the respondents.

Number	Lotteries	
Losses		
1	0.25,-1000	0.75,-100
2	0.4,-1000	0.6,0
3	0.1,-1000	0.9,-350
4	0.5,-1000	0.5,-250
5	0.5,-500	0.5,0
6	0.75,-900	0.25,-200
7	0.5,-750	0.5,-150
8	0.25,-750	0.75,0
Gains		
9	0.25,2000	0.75,200
10	0.4,2000	0.6,0
11	0.1,2000	0.9,700
12	0.5,2000	0.5,500
13	0.5,1000	0.5,0
14	0.75,1800	0.25,400
15	0.5,1500	0.5,300
16	0.25,1500	0.75,0
Mixed		
17	0.5,-X	0.5, 500
WTP		
18	4,000, $50,000	3,000, $X

### Analysis

The parameters of Eqs 1 and 2 were estimated by nonlinear regression [23]. The gain parameters α and γ were estimated simultaneously using the responses to questions 9–16 (from Tables 1 and 2). The same was done for the loss parameters β and δ with the responses to questions 1–8 (from Tables 1 and 2). Finally, the loss aversion coefficient λ was assessed by means of the indifference value obtained from the responses in the mixed prospect together with the other parameters obtained. Appendix A gives a derivation.

WTP values were estimated by plugging the indifference values of the WTP question into the following equations, for timeliness and FPR, respectively:

$$\mathrm{Timeliness}:\frac{\$X-\$50,000}{20-10};\;\mathrm{FPR}:\frac{\$Y-\$50,000}{4,000-3,000}.$$ (3)

Where the first one gives the WTP per day gained in detection and the second gives the WTP per 1/10,000 reduction in FPR.

In order to test the reliability of the data, we computed the number of violations of extended monotonicity. That is, because several lotteries were dominating others, the corresponding certainty equivalent should be higher for the dominating lotteries than for the dominated lotteries. Otherwise, a respondent would violate transitivity. For example, if we compare Lotteries 5 and 7 of Table 1, we can see that Lottery 7 will always result in a higher loss than Lottery 5. Hence, according to monotonicity, respondents will have to give a higher certainty equivalent (i.e. less negative) to Lottery 5. Considering monotonicity to be a fair criterion most subjects would like to satisfy, we have a measure of reliability of our data by counting the number of opposite cases.

## Results

The average rate of monotonicity violation is 4.5%. We checked for the presence of heterogeneous responses in our sample, by country of origin and country GDP, and there was no significant difference in any of the parameters of interest.

**Fig 1:**
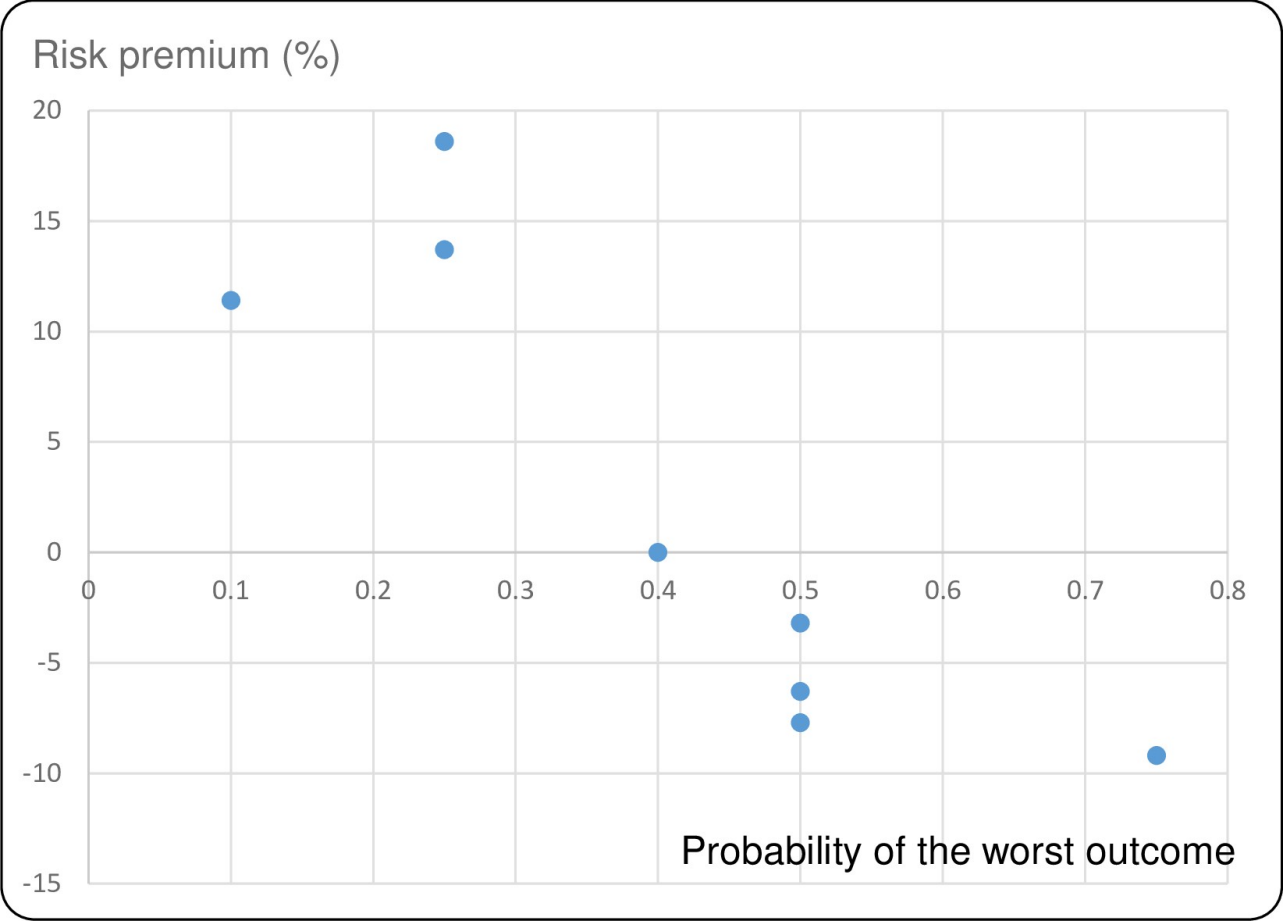
Risk premiums plotted against probability of the worst outcome in the losses lottery for timeliness.

**Fig 2:**
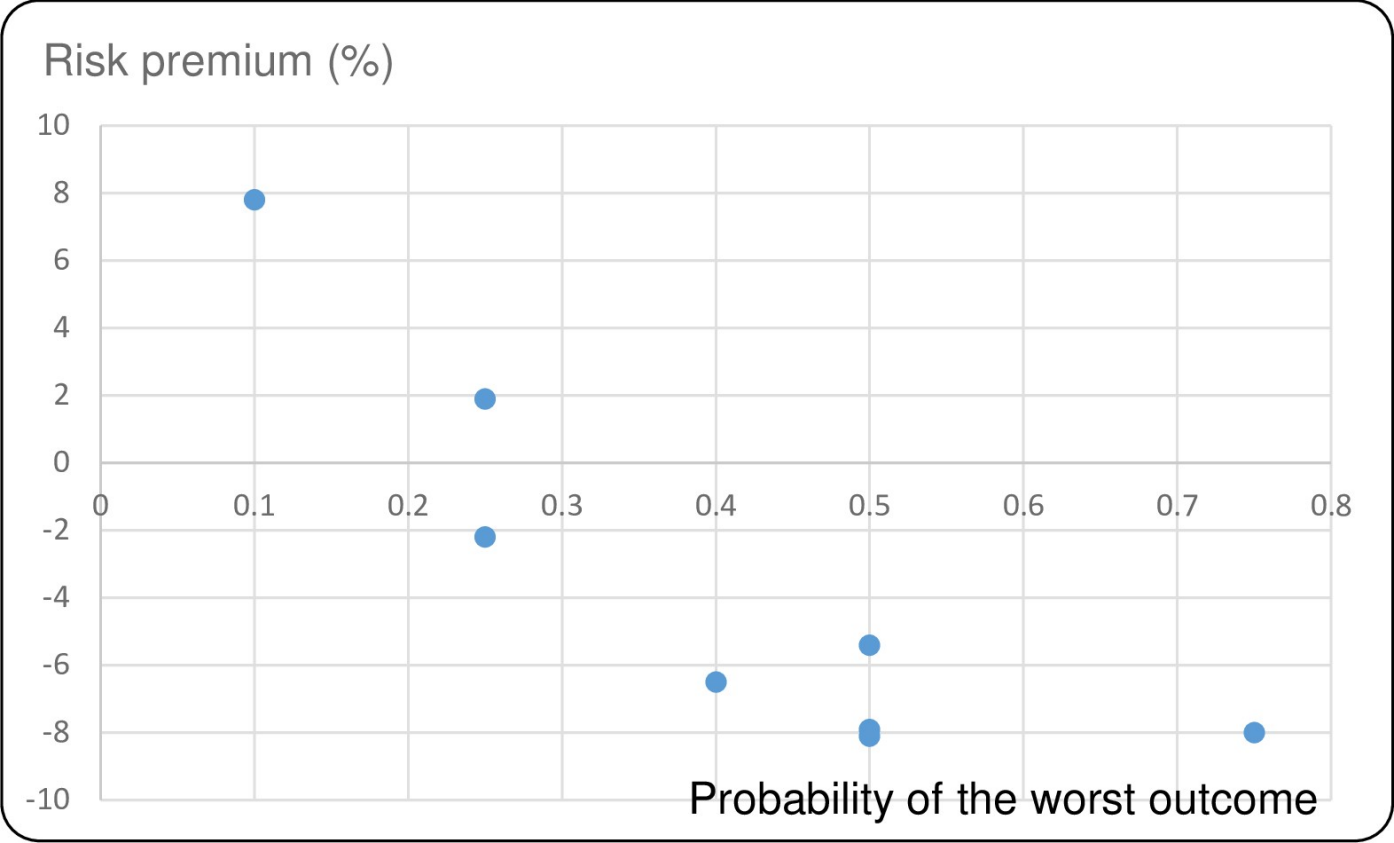
Risk premiums plotted against probability of the worst outcome in the losses lottery for FPR.

**Fig 3:**
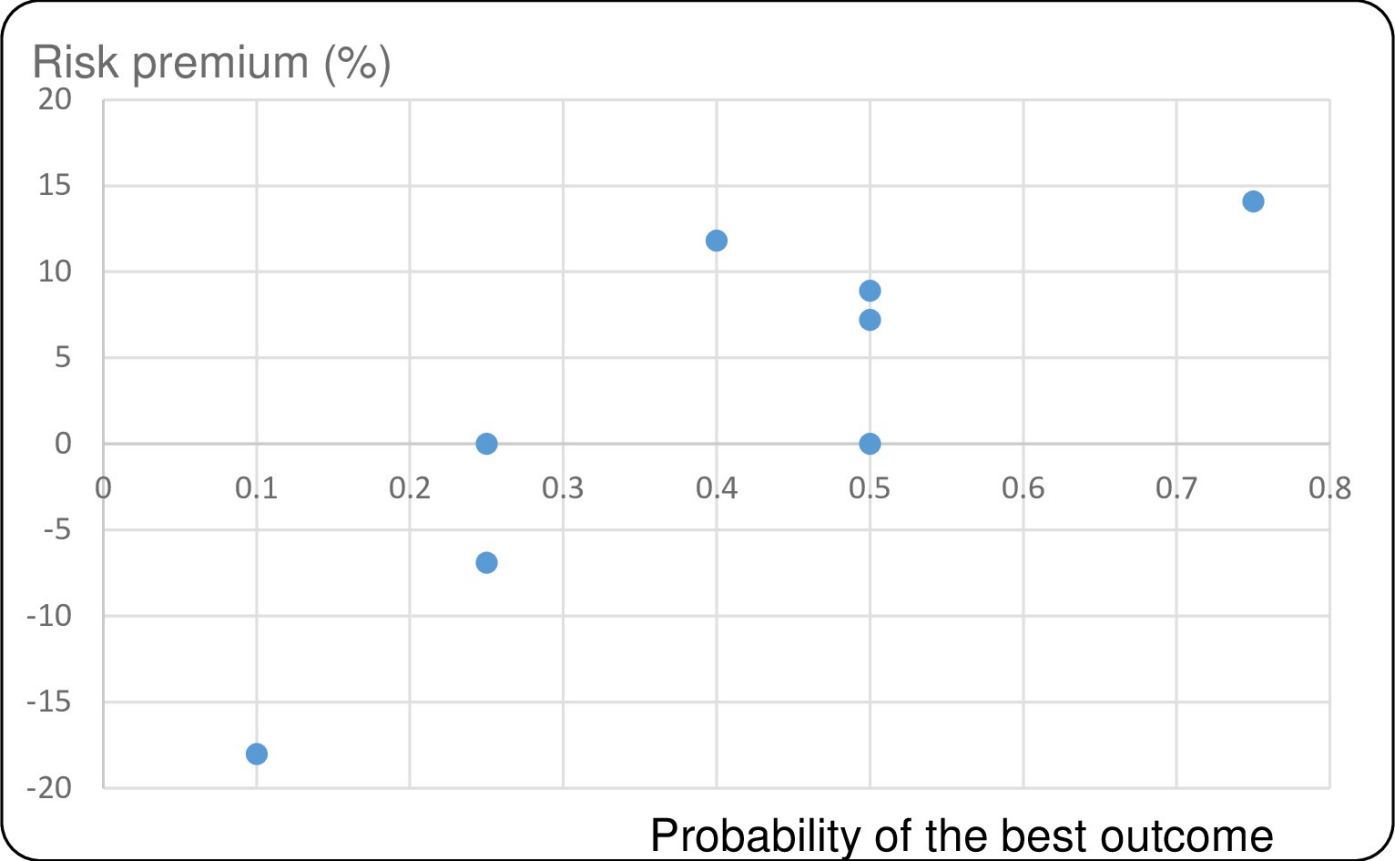
Risk premiums plotted against probability of the best outcome in the gains lottery for timeliness.

**Fig 4:**
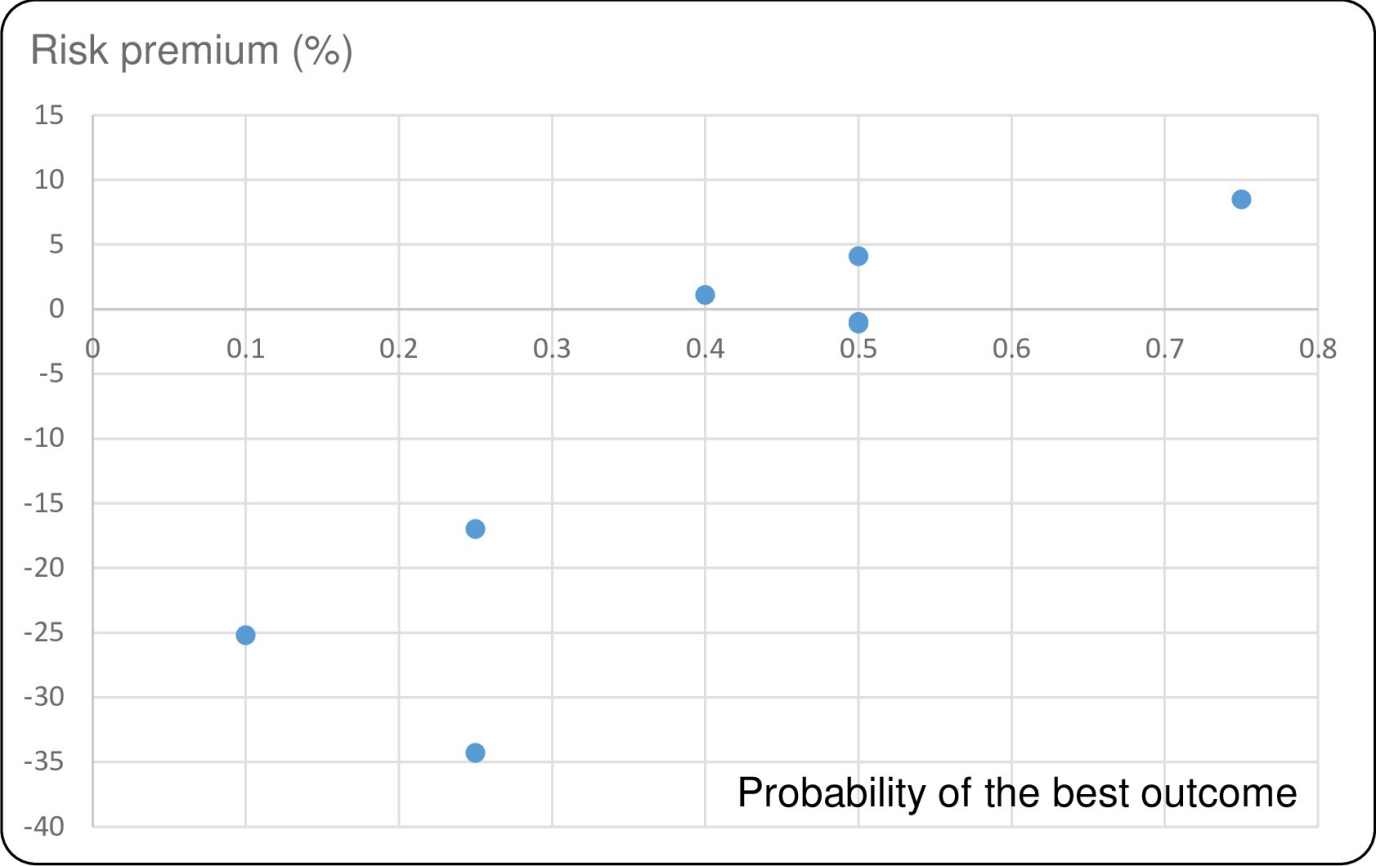
Risk premiums plotted against probability of the best outcome in the gains lottery for FPR.

Figs 1–4 show the average risk premiums, computed as (EV-CE)/EV for gains and (CE-EV)/EV for losses, where EV refers to the expected value of the lottery. This ratio could be read as a global measure of risk aversion. The higher this ratio, the greater is risk aversion. Figs 1 and 2 make clear that, for losses, there is a tendency to be risk averse (RA) for small probabilities of large losses and to become risk seeking (RS, corresponding to a negative risk premium) when these probabilities grow larger. For gains we observe risk seeking choices for small probabilities of the best outcome, and risk aversion for higher probabilities of this best outcome (Figs 3 and 4). This pattern is in agreement with a usual finding for decision making under risk, which is known as the *fourfold pattern of risk* [16].

For the mixed lotteries, we observe an average loss of X = 8.15 days for timeliness and 414/10,000 for FPR, which both indicate risk aversion, since these values are lower than the corresponding gains. That is, since the CE in the mixed lotteries was set at the reference point, risk neutrality [aversion, seeking] would require the absolute value of the elicited loss to be equal to [lower than, higher than] the gain. Tables B1-B4 in Appendix B shows more detailed statistics, including the average CEs for each lottery.

**Fig 5:**
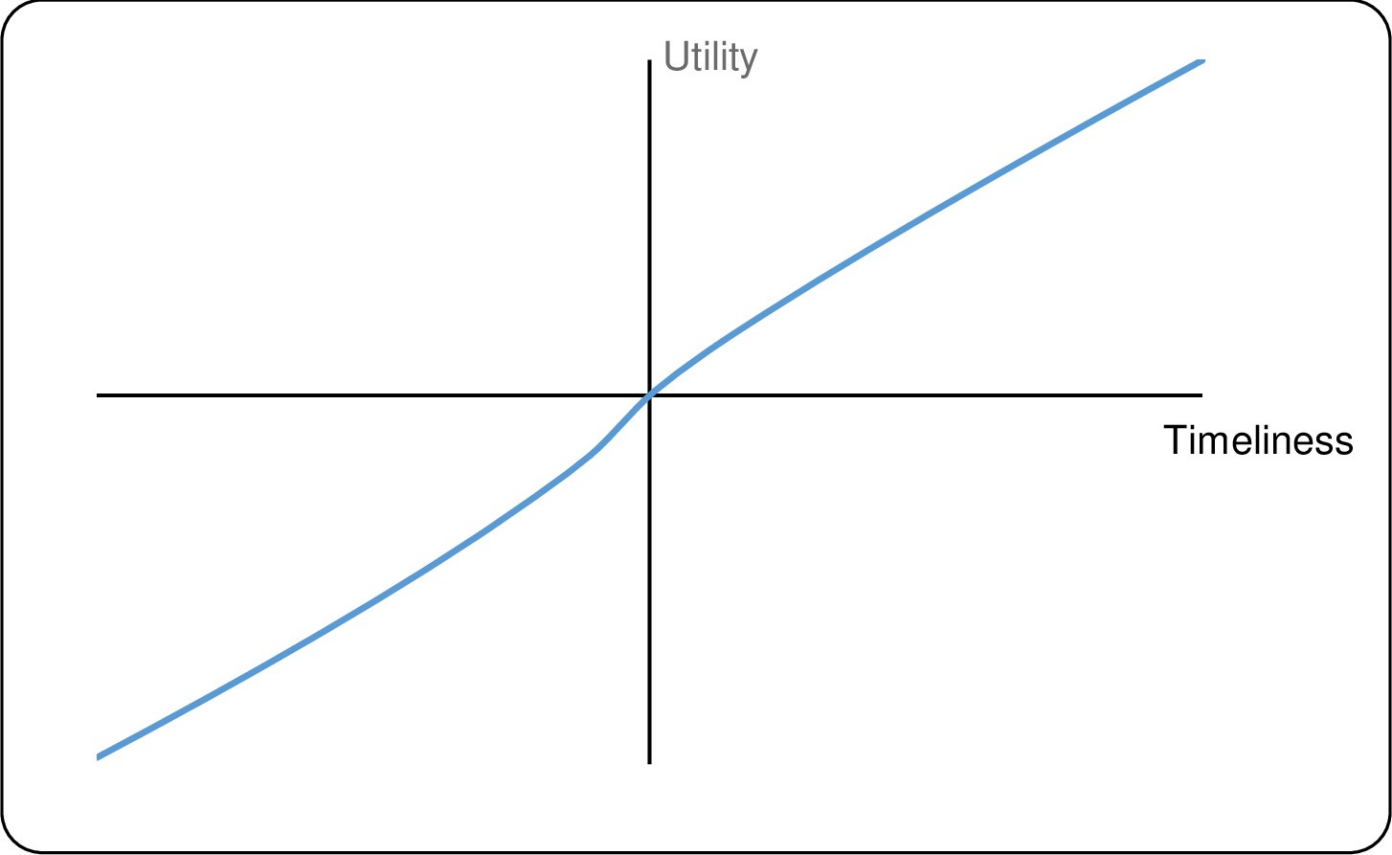
Plot of median utility (gains and losses) for timeliness.

**Fig 6:**
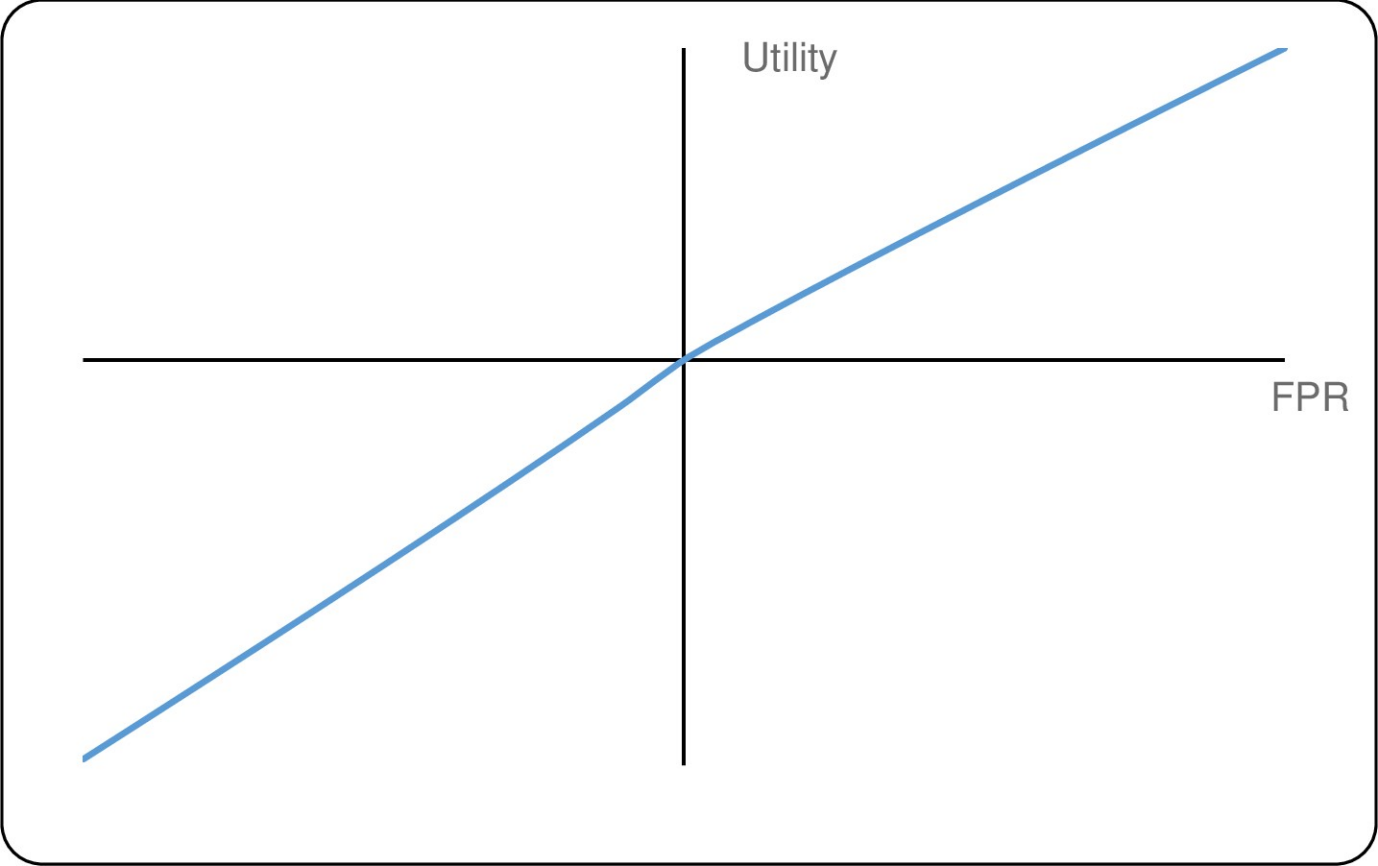
Plot of median utility (gains and losses) for FPR.

Table 3 gives the median and interquartile ranges of the parameter estimates for both outcomes. Fig 5 (timeliness) and Fig 6 (FPR) plot the utility functions corresponding to these medians utility parameters.

**Table 3:** Medians and interquartile ranges (IQR) of parameter estimates for both outcomes.

	*α*	*β*	*γ*	*δ*	λ
**Timeliness**					
Median	0.90	0.81	0.65	0.73	1.08
IQR	0.48–1.27	0.64–1.31	0.28–0.88	0.38–1.01	0.28–5.10
**FPR**					
Median	0.96	0.96	0.71	0.85	1.28
IQR	0.90–1.23	0.81–1.05	0.45–0.89	0.56–1.02	0.59–7.12

The power estimates were not significantly different from 1 (linearity). The estimates of the Prelec function provide convincing evidence in favor of an inverse-S shaped probability weighting function. These estimates are significantly lower than 1 for both attributes, and for both gains and losses (p<0.01 for all). Finally, we find slight evidence for loss aversion, although the medians are close to, and not significantly different from, 1 (p>0.13). The interquartile ranges make clear there is a lot of heterogeneity for the loss aversion index, which is again a common finding in the literature [21].

Comparison of the parameters between attributes revealed no differences (Mann-Whitney U-tests, all p’s>0.12). We also compared estimates of gains and losses, but these were not significantly different (Wilcoxon signed ranks tests, p>0.10).

In an additional analysis, we estimated the loss aversion coefficients for both outcomes assuming linear utility and no probability weighting. That is, we forced α = β = 1 and γ = δ = 1 and, hence, only estimated λ. This analysis gives significant loss aversion for both attributes (p<0.03), indicating that the loss aversion index is now picking up the influence of utility curvature and probability weighting. This stresses the importance of correcting for utility and probability weighting, because otherwise one would erroneously conclude that the risk aversion in the mixed prospect is completely caused by loss aversion.

### Willingness to pay

The median WTP for one day improvement (or a 5% out of the 20-day baseline) in timeliness is $7,250 (IQR $3,000-$10,000) and the median WTP for is $30 for a 0.01% improvement in FPR (IQR $18.75-$86.25). Hence, when assuming WTP per 0.01 percentage point improvement in FPR is constant irrespective of the amount of FPR, the median WTP for a 500 improvement (a 5% out of 10,000) in the FPR is 500*$30 = $15,000 (IQR $9,375-$43,125). In other words, respondents were 2.1 times more willing to pay for improvements in FPR than for those in timeliness.

### Reference points

**Fig 7:**
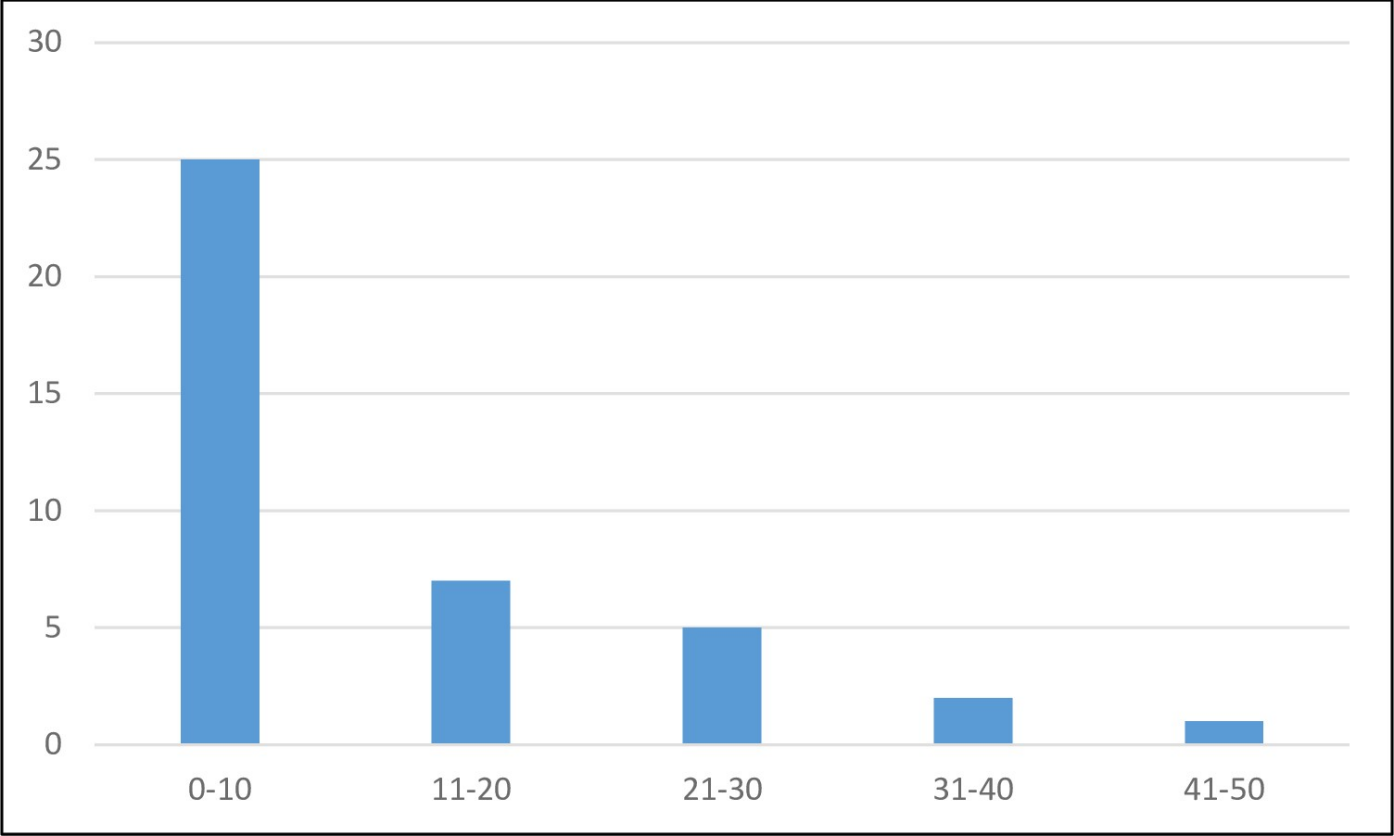
Bar chart of reference point for timeliness in days.

**Fig 8:**
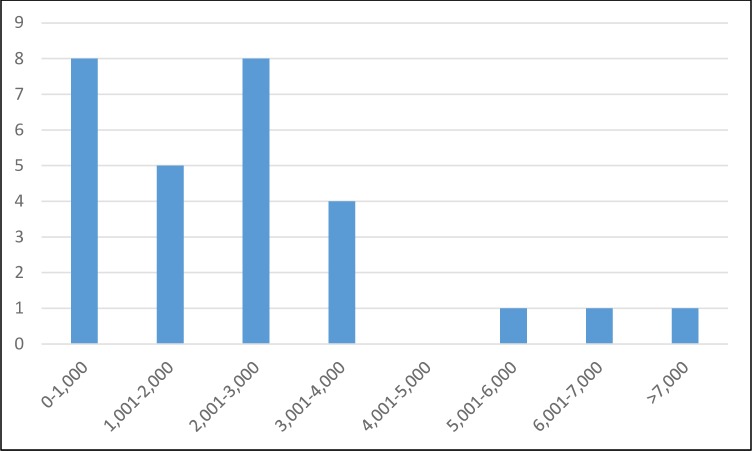
Bar chart of reference point for FPR (per 10,000).

Figs 7 and 8 show the distribution of the respondent’s own reference points. Taking the middle point of the five timeliness classes (1 to 10 days, 11 to 20, etc.), the average timeliness was 11.94 days. For FPR, the average from 28 respondents was 2,321/10,000 (setting a value of 7,500 for the respondent checking the >7000 box).

## Discussion

Our results show that respondents were risk averse due to probability weighting instead of concave utility of timeliness or FPR. A linear utility would not be surprising for rabies, given its almost inevitable fatal outcome and the importance of prompt detection of suspect rabid exposures to implement immediate PEP. For a rabies program facing budget cuts, the dis-utility perceived by the respondents in the face of de-investments in timeliness and FPR would also appear almost constant across the loss domain.

We also report loss aversion, which indicates that our respondents would perceive the dis-utility of reducing rabies capabilities as greater than the utility of similar gains. This might contribute to maintain rabies capacities and, specifically, the sustainability of the program by keeping the number of false positive cases low and hence limiting the prescription of expensive PEP. However, we should be cautious with drawing conclusions here, given that these results were not significant.

For gains, respondents were pessimistic about the probability of achieving the best return on their investment to improve timeliness and FPR. This might lead to underinvestment in timeliness and FPR once the program reaches a certain capacity level. This could hamper the current global rabies elimination efforts that will require enhanced detection to ensure prompt PEP application. Similarly, for programs with a certain capacity level, failure to invest to improve FPR would lead to sustained numbers of false positives and excessive PEP prescription.

An indication of the relative relevance of the two outcomes can be obtained from the difference in WTP. Respondents were keen to invest a significant dollar value for improvements in FPR. This may be explained by the fact that over 50% of the respondents to this question had FPR > 20%, leading to substantial PEP waste. However, this appetite was much reduced when facing improvements in timeliness. The latter may reflect the composition of our respondents, whom by large appeared to have timely surveillance systems.

A further study should assess whether behavior may be different depending on rabies risk, e.g. endemic vs. rabies free, in the country/area of the respondents. If behaviors in this context are to follow those for lotteries, changes in background risk could lead to changes in risk aversion [24].

The magnitude of the improvement (deterioration) after investments (de-investments) in timeliness and FPR is subject to uncertainty. However, one would logically expect improvements after investments, and deterioration after de-investments. The standard example in health settings describes the deployment of some treatment that, while improving health, could also lead to adverse effects. Such adverse effects in our surveillance setting, from the improvement of our targeted surveillance capacities, seem difficult to describe. Possible deterioration of timeliness after investment could stem from faulty implementation. For example, the expectation of active tracings by exposed people following a bite incident, and the failure of the authorities to deliver such service, could lead to the assumption by the truly exposed that dog rabies was not confirmed and they need no PEP. This could lead to fatal delays. Similarly, timeliness could be negatively impacted, even after investments to its improvement, as a result of consequences following measures considered too harsh by the affected community. For example, active tracings to improve timeliness could lead to an increase in the number of euthanized dogs. This could lead to failure to report bites in the first place. More commonly in our surveillance setting, the equivalent of possible adverse effects following investment in timeliness might come from the increase in the number of false positives. Similarly, an improvement in the FPR, for example by delaying PEP application after observation of suspect dogs, could result in a deterioration of timeliness. This type of dependence between outcomes is not contemplated in this work.

Regardless of the source or mechanisms leading to negative outcomes, our research highlights the need to assess all possible scenarios during the planning and comparison of alternative surveillance interventions. Economic evaluations should explore the prospect of interventions leading to negative outcomes and the likely occurrence of loss aversion. In such situations, effectiveness models should adjust for such artefacts.

### Applications

The objective of the work was to evaluate the presence of PT cognitive biases in surveillance investment decisions. In other words, our results inform strategic settings. They do not aim to change or inform the clinical management of rabies cases and hence our target are not frontline health care staff, but budget and portfolio managers who need to assess the utility of different surveillance alternatives, for rabies in this instance.

Rabies surveillance is fairly unrefined. Only recently there have been efforts to evaluate the efficiency of its different forms, for example, of the integrated bite case management [25]. This form of surveillance entails active contact tracing and application of PEP to suspect exposures. Our results could expand on the economic evaluation of different rabies surveillance alternatives, e.g. passive surveillance vs. active surveillance, by plotting their incremental cost effectiveness ratio (ICER) against our elicited process-specific WTP. At this point, we note that our outcomes, timeliness and FPR, are process-related and diverge from the outcomes, e.g. quality-adjusted life years (QALYs), deaths averted, normally targeted in health economic evaluations.

A further application of our results is in the extension of probabilistic sensitivity analyses to include our computed risk aversion parameter. Briefly, the specification of the utility function, for each surveillance alternative and outcome in our case, as a net benefit assumes a risk-neutral decision maker. Adjustments to include risk aversion are possible [26], and desirable, that would return unbiased estimates of the comparators of interest (e.g. expected incremental benefit) between the surveillance alternatives. Given the little appetite for inefficiencies in the last mile of disease control and elimination programs [4], fine-tuned comparisons between the multiple interventions, taking into account the behavioral biases here elicited, is a must.

### Limitations and perspectives

We could not implement the RPs of the respondents to inform our lotteries. This would have required dynamic adjustment of the lotteries as the respondents completed the questionnaire. Given the online nature of the exercise, this was not feasible. The results of the reference point estimation suggest that the reference points we implemented in our lottery questions were too high (i.e. 20 days vs. an average of 11.75 days for timeliness and 4,000/10,000 vs. 2,321/10,000). This implies that, if respondents took their own estimate as reference point instead of the one given in the instructions to the lottery questions, they were on average more likely to consider outcomes as losses than we assumed. However, our findings of concave utility for gains and convex utility for losses (as seen from the reference points used in the S1 Instructions questionnaire), as well as the evidence of loss aversion, indicate that these induced reference points were indeed adopted in our choice tasks. Such a finding was also reported by related studies [27,28].

No validation workshop was possible given the widespread location of our respondents. We focused on timeliness and FPR. Other surveillance attributes could have been targeted, such as sensitivity. However, given the cognitive burden on respondents, and the *a priori* uncertain number of them, we decided to restrict our scope to two attributes.

Our target population was made of public health and animal health professionals. In other words, a homogenous highly educated group that should lead to no differences between their responses as seen in related studies assessing risks [29] due to variations in numeracy. We however recognize that lottery questions do present a significant cognitive burden on respondents (this was raised by some of our respondents who expressed the difficulty in addressing the questions). Still, the dominance violation rate of 4.5% may be considered fairly low, given that we should allow for response errors and imprecise preferences. Although our analyses did not find any difference within our cohort, it may be possible that greater probability weighing biases may occur in populations with lower numerical skills [29]. Our choice of networks and approach to polling them was a convenient one. We did not seek to poll rabies experts but a cohort of knowledgeable health professionals to test whether they might display PT-specific cognitive biases around the selection of surveillance alternatives. Although one third of the participants had not worked on rabies, we considered that their experience on DSS and One Health issues qualified them to understand the purpose of the experiment and aptly complete the questionnaire. We stress that our results should be interpreted cautiously, and we recommend further research on this topic using a larger sample size to be able to test for differences in results between different demographic groups.

## Supporting information

S1 Raw data Raw dataset. (CSV)

S1 Text Appendix A.
(DOCX)

S2 Text Appendix B.
(DOCX)

S1 Table Loss lotteries timeliness. EV stands for expected value, CE for certainty equivalent. Risk attitudes are risk neutral (RN) when EV = CE, risk averse (RA) when EV>CE, and risk seeking (RS) when EV<CE.
(DOCX)

S2 Table Loss lotteries FPR. EV stands for expected value, CE for certainty equivalent. Risk attitudes are risk neutral (RN) when EV = CE, risk averse (RA) when EV>CE, and risk seeking (RS) when EV<CE.
(DOCX)

S3 Table Gain lotteries timeliness. EV stands for expected value, CE for certainty equivalent. Risk attitudes are risk neutral (RN) when EV = CE, risk averse (RA) when EV>CE, and risk seeking (RS) when EV<CE.
(DOCX)

S4 Table Gain lotteries FPR. EV stands for expected value, CE for certainty equivalent. Risk attitudes are risk neutral (RN) when EV = CE, risk averse (RA) when EV>CE, and risk seeking (RS) when EV<CE.
(DOCX)

S5 Table List of respondents’ country of origin. (DOCX)

S6 Table Demographic data. (DOCX)
