# Supplementary material for: Unbiased assessment of disease surveillance utilities: A prospect theory application
Source: PLoS Negl Trop Dis. 2019 May 1;13(5):e0007364. doi: 10.1371/journal.pntd.0007364 (PMC6513105; doi:10.1371/journal.pntd.0007364)
Supplement: S1 Text — Appendix A. (DOCX) [file pntd.0007364.s002.docx]

**Appendix A. Derivations of formulas**

Using functions (1) and (2), the utility of gain prospects were evaluated as follows:

$\left( exp\left\{ -\left( -ln(p) \right)^{\gamma} \right\} \right)\times\left( x^{\alpha}-y^{\alpha} \right)+y^{\alpha}$. (A1)

Similarly, for loss prospects we get:

$\left( exp\left\{ -\left( -ln(p) \right)^{\delta} \right\} \right)\times\left( -(-{x)}^{\beta}+(-{y)}^{\beta} \right)-(-{y)}^{\beta}$. (A2)

We assume that observable utility U is a composition of a loss aversion coefficient λ and a basic utility u [15]:

$U\left( x \right)=\left\{ \begin{aligned} u\left( x \right) if x\geq0 \\ \lambda u\left( x \right)if x<0 \end{aligned} \right.$. (A3)

In Eq. A3, a decision maker is said to be loss averse if λ>1, gain seeking if λ<1, and loss neutral if λ=1. As a result of Eqs. 1, 2 and A3, the evaluation for mixed prospects becomes:

$\left( exp\left\{ -\left( -ln(p) \right)^{\gamma} \right\} \right)\times x^{\alpha}+\lambda$ $\times\left( exp\left\{ -\left( -ln(1-p) \right)^{\delta} \right\} \right)\times{-(-y)}^{\beta}$. (A4)

For gains, an indifference is evaluated by:

${z^{+}}^{\alpha}=\left( exp\left\{ -\left( -ln(p) \right)^{\gamma} \right\} \right)\times\left( x^{\alpha}-y^{\alpha} \right)+y^{\alpha}$. (A5)

We can solve this equation for $z^{+}$ to obtain the regression equation:

$z^{+}=\left[ \left( exp\left\{ -\left( -ln(p) \right)^{\gamma} \right\} \right)\times\left( x^{\alpha}-y^{\alpha} \right)+y^{\alpha} \right]^{1/\alpha}$. (A6)

Likewise, for losses we get:

$z^{-}=\left[ \left( exp\left\{ -\left( -ln(p) \right)^{\delta} \right\} \right)\times\left( {-(-x)}^{\beta}+{(-y)}^{\beta} \right)-(-{y)}^{\beta} \right]^{1/\beta}$. (A7)

Setting equation A4 equal to 0:

$\left( exp\left\{ -\left( -ln(p) \right)^{\gamma} \right\} \right)\times x^{\alpha}+$ $\lambda\left( exp\left\{ -\left( -ln(1-p) \right)^{\delta} \right\} \right)\times y^{\beta}$=0. (A8)

Solving (A8) for λ yields:

$\lambda=-\frac{\left( exp\left\{ -\left( -ln(p) \right)^{\gamma} \right\} \right)\times x^{\alpha}}{\left( exp\left\{ -\left( -ln(1-p) \right)^{\delta} \right\} \right)\times-(-{y)}^{\beta}}$. (A9)

Now, $\lambda$ can be computed by inserting the four gain and loss parameter estimates, together with the relevant values of p, x and y, into Eq. A8.
