# Supplementary material for: Unbiased assessment of disease surveillance utilities: A prospect theory application
Source: PLoS Negl Trop Dis. 2019 May 1;13(5):e0007364. doi: 10.1371/journal.pntd.0007364 (PMC6513105; doi:10.1371/journal.pntd.0007364)
Supplement: S2 Text — Appendix B. (DOCX) [file pntd.0007364.s003.docx]

**Appendix B. Additional results**

S1 Table. Loss lotteries timeliness. EV stands for expected value, CE for certainty equivalent. Risk attitudes are risk neutral (RN) when EV=CE, risk averse (RA) when EV>CE, and risk seeking (RS) when EV<CE.

S2 Table. Loss lotteries FPR. EV stands for expected value, CE for certainty equivalent. Risk attitudes are risk neutral (RN) when EV=CE, risk averse (RA) when EV>CE, and risk seeking (RS) when EV<CE.

S3 Table. Gain lotteries timeliness. EV stands for expected value, CE for certainty equivalent. Risk attitudes are risk neutral (RN) when EV=CE, risk averse (RA) when EV>CE, and risk seeking (RS) when EV<CE.

S4 Table. Gain lotteries FPR. EV stands for expected value, CE for certainty equivalent. Risk attitudes are risk neutral (RN) when EV=CE, risk averse (RA) when EV>CE, and risk seeking (RS) when EV<CE.

S5 Table. List of respondents’ country of origin.

S6 Table. Demographic data.
