# Supplementary material for: Unbiased assessment of disease surveillance utilities: A prospect theory application
Source: PLoS Negl Trop Dis. 2019 May 1;13(5):e0007364. doi: 10.1371/journal.pntd.0007364 (PMC6513105; doi:10.1371/journal.pntd.0007364)
Supplement: S1 Table — EV stands for expected value, CE for certainty equivalent. Risk attitudes are risk neutral (RN) when EV = CE, risk averse (RA) when EV>CE, and risk seeking (RS) when EV<CE. (DOCX) [file pntd.0007364.s004.docx]

*Loss lotteries timeliness. EV stands for expected value, CE for certainty equivalent. Risk attitudes are risk neutral (RN) when EV=CE, risk averse (RA) when EV>CE, and risk seeking (RS) when EV<CE.*

| Prospect | Lotteries | | EV | Average CE | Risk premium | Risk Attitude |
| --- | --- | --- | --- | --- | --- | --- |
| 1 | 0.25,-70 | 0.75,-7 | -22.75 | -26.99 | 18.6% | RA |
| 2 | 0.4,-70 | 0.6,0 | -28 | -27.95 | 0% | RN |
| 3 | 0.1,-70 | 0.9,-25 | -29.5 | -32.87 | 11.4% | RA |
| 4 | 0.5,-70 | 0.5,-18 | -44 | -42.59 | -3.2% | RS |
| 5 | 0.5,-35 | 0.5,0 | -17.5 | -16.4 | -6.3% | RS |
| 6 | 0.75,-63 | 0.25,-14 | -50.75 | -46.10 | -9.2% | RS |
| 7 | 0.5,-53 | 0.5,-11 | -32 | -29.54 | -7.7% | RS |
| 8 | 0.25,-53 | 0.75,0 | -13.25 | -15.07 | 13.7% | RA |
