# Supplementary material for: Unbiased assessment of disease surveillance utilities: A prospect theory application
Source: PLoS Negl Trop Dis. 2019 May 1;13(5):e0007364. doi: 10.1371/journal.pntd.0007364 (PMC6513105; doi:10.1371/journal.pntd.0007364)
Supplement: S2 Table — EV stands for expected value, CE for certainty equivalent. Risk attitudes are risk neutral (RN) when EV = CE, risk averse (RA) when EV>CE, and risk seeking (RS) when EV<CE. (DOCX) [file pntd.0007364.s005.docx]

*Loss lotteries FPR. EV stands for expected value, CE for certainty equivalent. Risk attitudes are risk neutral (RN) when EV=CE, risk averse (RA) when EV>CE, and risk seeking (RS) when EV<CE.*

| Prospect | Lotteries | | EV | Average CE | Risk premium | Risk Attitude |
| --- | --- | --- | --- | --- | --- | --- |
| 1 | 0.25,-1000 | 0.75,-100 | -325 | -317.86 | -2.2% | RS |
| 2 | 0.4;-1000 | 0.6,0 | -400 | -374.11 | -6.5% | RS |
| 3 | 0.1;-1000 | 0.9,-350 | -415 | -447.32 | 7.8% | RA |
| 4 | 0.5,-1000 | 0.5,-250 | -625 | -574.11 | -8.1% | RS |
| 5 | 0.5,-500 | 0.5,0 | -250 | -236.61 | -5.4% | RS |
| 6 | 0.75,-900 | 0.25,-200 | -725 | -666.96 | -8% | RS |
| 7 | 0.5,-750 | 0.5,-150 | -450 | -414.29 | -7.9% | RS |
| 8 | 0.25,-750 | 0.75,0 | -187.5 | -191.07 | 1.9% | RA |
