# Supplementary material for: Unbiased assessment of disease surveillance utilities: A prospect theory application
Source: PLoS Negl Trop Dis. 2019 May 1;13(5):e0007364. doi: 10.1371/journal.pntd.0007364 (PMC6513105; doi:10.1371/journal.pntd.0007364)
Supplement: S3 Table — EV stands for expected value, CE for certainty equivalent. Risk attitudes are risk neutral (RN) when EV = CE, risk averse (RA) when EV>CE, and risk seeking (RS) when EV<CE. (DOCX) [file pntd.0007364.s006.docx]

*Gain lotteries timeliness. EV stands for expected value, CE for certainty equivalent. Risk attitudes are risk neutral (RN) when EV=CE, risk averse (RA) when EV>CE, and risk seeking (RS) when EV<CE.*

| Prospect | Lotteries | | EV | Average CE | Risk premium | Risk Attitude |
| --- | --- | --- | --- | --- | --- | --- |
| 1 | 0.25,20 | 0.75,2 | 6.5 | 6.55 | 0% | RN |
| 2 | 0.4,20 | 0.6,0 | 8 | 7.06 | 11.8% | RA |
| 3 | 0.1,20 | 0.9,7 | 8.3 | 9.79 | -18% | RS |
| 4 | 0.5,20 | 0.5,5 | 12.5 | 11.39 | 8.9% | RA |
| 5 | 0.5,10 | 0.5,0 | 5 | 5 | 0% | RN |
| 6 | 0.75,18 | 0.25,4 | 14.5 | 12.45 | 14.1% | RA |
| 7 | 0.5,15 | 0.5,3 | 9 | 8.35 | 7.2% | RA |
| 8 | 0.25,15 | 0.75,0 | 3.75 | 4.01 | -6.9% | RS |
