# Supplementary material for: Unbiased assessment of disease surveillance utilities: A prospect theory application
Source: PLoS Negl Trop Dis. 2019 May 1;13(5):e0007364. doi: 10.1371/journal.pntd.0007364 (PMC6513105; doi:10.1371/journal.pntd.0007364)
Supplement: S4 Table — EV stands for expected value, CE for certainty equivalent. Risk attitudes are risk neutral (RN) when EV = CE, risk averse (RA) when EV>CE, and risk seeking (RS) when EV<CE. (DOCX) [file pntd.0007364.s007.docx]

*Gain lotteries FPR. EV stands for expected value, CE for certainty equivalent. Risk attitudes are risk neutral (RN) when EV=CE, risk averse (RA) when EV>CE, and risk seeking (RS) when EV<CE.*

| Prospect | Lotteries | | EV | Average CE | Risk premium | Risk Attitude |
| --- | --- | --- | --- | --- | --- | --- |
| 1 | 0.25,2000 | 0.75,200 | 650 | 760.71 | -17% | RS |
| 2 | 0.4,2000 | 0.6,0 | 800 | 791.07 | 1.1% | RA |
| 3 | 0.1,2000 | 0.9,700 | 830 | 1039.3 | -25.2% | RS |
| 4 | 0.5,2000 | 0.5,500 | 1250 | 1198.2 | 4.1% | RA |
| 5 | 0.5,1000 | 0.5,0 | 500 | 505.36 | -1.1% | RN |
| 6 | 0.75,1800 | 0.25,400 | 1450 | 1326.8 | 8.5% | RA |
| 7 | 0.5,1500 | 0.5,300 | 900 | 908.93 | -1.0% | RN |
| 8 | 0.25,1500 | 0.75,0 | 375 | 503.57 | -34.3% | RS |
