# Supplementary material for: Unbiased assessment of disease surveillance utilities: A prospect theory application
Source: PLoS Negl Trop Dis. 2019 May 1;13(5):e0007364. doi: 10.1371/journal.pntd.0007364 (PMC6513105; doi:10.1371/journal.pntd.0007364)
Supplement: S5 Table — (DOCX) [file pntd.0007364.s008.docx]

*List of respondents’ country of origin*

| **Country** | **Number of respondents** |
| --- | --- |
| Austria | 2 |
| Australia | 5 |
| Belgium | 1 |
| Canada | 4 |
| Chad | 1 |
| China | 1 |
| Denmark | 2 |
| France | 1 |
| India | 2 |
| Iran | 1 |
| Ireland | 1 |
| Italy | 2 |
| Jamaica | 1 |
| Kenya | 1 |
| Madagascar | 1 |
| Mexico | 1 |
| Netherlands | 3 |
| Nigeria | 1 |
| Philippines | 2 |
| Sweden | 1 |
| Switzerland | 2 |
| United Kingdom | 4 |
| United States of America | 27 |
