# Supplementary material for: Unbiased assessment of disease surveillance utilities: A prospect theory application
Source: PLoS Negl Trop Dis. 2019 May 1;13(5):e0007364. doi: 10.1371/journal.pntd.0007364 (PMC6513105; doi:10.1371/journal.pntd.0007364)
Supplement: S6 Table — (DOCX) [file pntd.0007364.s009.docx]

*Demographic data*

**Rabies-free countries**

**Age group**

| **Gender** | **20-29** | **30-39** | **40-49** | **50-59** | **60-69** | **Total** |
| --- | --- | --- | --- | --- | --- | --- |
| **Male** | 0 | 0 | 3 | 4 | 3 | 10 |
| **Female** | 1 | 9 | 6 | 3 | 0 | 19 |
| **Total** | 1 | 9 | 9 | 7 | 3 | 29 |

**Rabies-endemic countries (including United States)**

**Age group**

| **Gender** | **20-29** | **30-39** | **40-49** | **50-59** | **60-69** | **Total** |
| --- | --- | --- | --- | --- | --- | --- |
| **Male** | 2 | 2 | 4 | 4 | 1 | 13 |
| **Female** | 4 | 9 | 3 | 8 | 1 | 25 |
| **Total** | 6 | 11 | 7 | 12 | 2 | 38 |

Source: [www.who.int](http://www.who.int)
